# Identification of the translational start site of codon-optimized mCherry in *Mycobacterium tuberculosis*

**DOI:** 10.1186/1756-0500-7-366

**Published:** 2014-06-17

**Authors:** Paul Carroll, Julian Muwanguzi-Karugaba, Eduard Melief, Megan Files, Tanya Parish

**Affiliations:** 1 Queen Mary University of London, Barts & The London School of Medicine and Dentistry, Centre for Immunology and Infectious Disease, London, UK; 2 Infectious Disease Research Institute, Seattle, Washington, USA

**Keywords:** Fluorescence, Gene expression, Mycobacteria, Reporter genes

## Abstract

**Background:**

Fluorescent proteins are used widely as reporter genes in many organisms. We previously codon-optimized mCherry for *Mycobacterium tuberculosis* and generated expression constructs with high level expression in mycobacteria with multiple uses *in vitro* and *in vivo*. However, little is known about the expression of fluorescent proteins in mycobacteria and the translational start codon for mCherry has not been experimentally determined.

**Results:**

We determined the translational start site for functional (fluorescent) mCherry in mycobacteria. Several potential translational start codons were identified; introduction of downstream stop codons by mutagenesis was used to determine which start codon was utilized in the bacterial cells. Fluorescent protein was expressed from a construct which would allow translation of a protein of 226 amino acids or a protein of 235 amino acids. No fluorescence was seen when a construct which could give rise to a protein of 219 amino acids was used. Similar results were obtained in mycobacteria and in *Escherichia coli*. Western blotting confirmed that mCherry was expressed from the constructs encoding 235 or 226 amino acids, but not from the plasmid encoding 219 amino acids. N-terminal sequencing and mass determination confirmed that the mature protein was 226 amino acids and commenced with the amino acid sequence AIIKE.

**Conclusion:**

We conclude that mCherry is expressed in *M. tuberculosis* as a smaller protein than expected lacking the GFP-derived N-terminal sequence designed to allow efficient fusions.

## Background

Fluorescent proteins (FPs), in particular GFP derivatives, are widely used in mycobacterial systems for many applications [1-13]. Numerous FP variants have been developed, many of which are variants of the original green fluorescent protein (GFP) from the jellyfish *Aequorea victoria *[3,14], or the red fluorescent protein DsRed from the coral *Discosoma* sp red [15,16]. The wide range of FPs available have different features, including different excitation and emission wavelengths, expression as monomers or dimers, stability, susceptibility to photobleaching, and responsiveness to environmental conditions such as pH or redox [6,17-22].

We are interested in the use of FPs as reporters in mycobacteria. We have previously generated FP variants with codon usage optimized for *Mycobacterium tuberculosis *[2,13]. We were able to generate plasmid expression systems with high level constitutive expression that resulted in highly fluorescent strains of use for both *in vitro* and *in vivo* studies [2,13]. We investigated the use of alternative promoters to generate the highest level of expression possible, without inducing plasmid instability or compromising bacterial growth or virulence [2,13]. Such reporter strains are of use in multiple applications, including gene expression studies [2], identifying novel anti-tubercular agents [12,23,24] or monitoring growth *in vivo *[13].

The level of expression of an FP is dependent on transcriptional and translational factors, but in bacteria the main factor is the promoter. The promoter location in relation to the transcriptional and translational start site can play a role in determining overall expression levels. In our original constructs we used the translational start site of the engineered protein mCherry from the literature in order to place the gene in the correct location relative to the promoter. The original development of mCherry involved replacing the first seven amino acids to improve the ability to generate functional N-terminal fusions, although the sequence still retained a downstream methionine which could function as an alternative start [17]. In order to generate further improved constructs and to characterize the recombinant fluorescent protein expressed in mycobacteria, we determined the functional translational start site for mCherry. Our data demonstrate that mCherry is expressed in *M. tuberculosis* as a truncated protein which lacks the N-terminal sequence derived from GFP (MVSKGEE).

## Results and discussion

We are interested in the use of FPs as reporters of bacterial viability and gene expression in mycobacteria, in particular in *M. tuberculosis *[2,13]. The expression of FPs at high level can be detrimental to bacterial viability due to the metabolic burden it places on the cell. We have developed codon-optimized FPs for *M. tuberculosis* to, at least partly, overcome this by removing rare codons and allowing for high level expression [2].

### The functional translational start site for mCherry in *M. tuberculosis*

**Figure 1:**
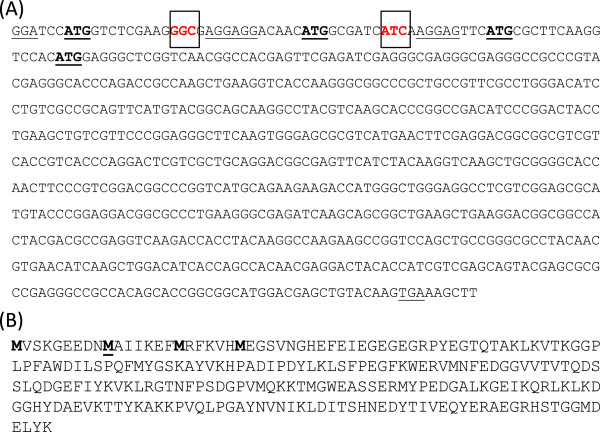
**Sequence of mCherry. (A)** DNA sequence of codon-optimized mCherry gene. The potential translational start sites are indicated in bold and underlined; potential ribosome-binding sites and the stop codon are underlined. The codons mutagenized to stop codons are in red and boxed. **(B)** Protein sequence of mCherry indicating the four potential start sites in bold. The identified start site is underlined.

We previously optimized expression of mCherry by codon-optimization of the most commonly used variant [2]. During our studies we noted that there were several potential translational start sites. Three of these (including the expected one) had a ribosome binding site (RBS) motif directly upstream, suggesting that translation could initiate in multiples places (Figure 1). Interestingly, the two internal RBS and the downstream start codons were present in the original sequence and were not introduced by codon optimization. The translational start site of *Ds*Red, from which mCherry is derived, has not been determined functionally for the native protein [15]. In the original studies the *Ds*Red protein was expressed with a His-Tag and the primary amino acid sequence was derived from the sequence of the mRNA rather than protein sequencing [15]. mCherry itself was constructed by replacing the first seven amino acids of mRFP1.1 with the corresponding GFP sequence and is thus a fusion protein. Therefore it is possible that the functional protein is shorter than that expected, or that different proteins could be produced in different species depending on the efficiency of the RBS and the translational machinery.

We decided to determine which of the alternative translational start sites are functional in *M. tuberculosis*. We used an expression construct (pCherry10) in which mCherry is under the control of the G13 promoter from *M. marinum*[13]; this is a strong constitutive promoter. There are three potential translational start sites at +1, +28 and +52 (relative to the expected start site); to determine which of these was functioning in mycobacteria, we introduced a stop codon either at +13 or at +40 (plasmids pCherry29 and pCherry30). This would prevent translation of full length protein from the +1 or +28 start sites respectively and translation would results in proteins of 226 or 219 amino acids, instead of 235 in the “full length” version.

**Figure 2:**
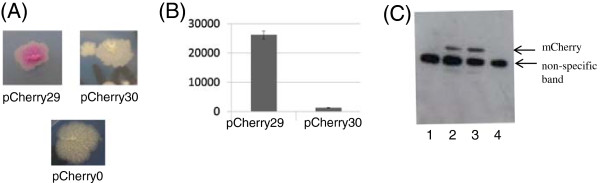
**Identification of the translational start site of mCherry in *M. tuberculosis*.** Plasmids carrying mCherry were electroporated into *M. tuberculosis* and transformants selected on solid medium using hygromycin. The predicted proteins expressed from each plasmid are - pCherry29 = mCherry_226_; pCherry30 = mCherry_219_; pCherry0 (control plasmid) = no mCherry expression. **(A)** Transformant colonies. **(B)** Fluorescence was measured in liquid culture. Cultures were measured at Ex587/Em610 and results are expressed as relative fluorescence units (fluorescence/OD). Data are the mean and standard deviation from three independent transformants **(C)** Western analysis of protein expression in *E. coli*. Cell-free extracts were generated from transformants carrying plasmids and probed with anti-mCherry antibodies. Lane 1: no plasmid. Lane 2- pCherry10 (mCherry_235_). Lane 3- pCherry29 (mCherry_226_). Lane 4- pCherry30 (mCherry_219_). A non-specific band reacting with the commercial antibody was seen in all lanes, including *E. coli* lacking a plasmid.

Constructs were introduced into *M. tuberculosis*. We already demonstrated that functional fluorescent protein was produced from the parental vector pCherry10 [13]. It was apparent immediately that functional FP was made from one of the expression vectors, since the colonies were visibly colored (Figure 2A); this was confirmed by measuring fluorescence in cells (Figure 2B). No difference in fluorescence intensity was seen when a stop codon was introduced upstream of the start codon at +28, whereas a complete loss of fluorescence was noted when a stop codon was introduced upstream of the +52 start codon. These data confirm that functional, fluorescent protein is obtained in *M. tuberculosis* from a truncated protein (mCherry_226_), but not from mCherry_219_.

The same results were obtained in *Escherichia coli* (data not shown), where fluorescence was seen with mCherry_226_, but not mCherry_219_. This suggested that the functional translational start site was the same in both species. In order to determine if the lack of fluorescence was from lack of expression or if the protein was produced, but not functional, we looked at protein levels by Western blotting. Expression of mCherry was seen from plasmid pCherry10 and pCherry29, but not from pCherry30, suggesting that the translational start site at +52 does not lead to the production of protein (Figure 2C).

### mCherry is expressed as a mature protein of 226 amino acids

**Figure 3:**
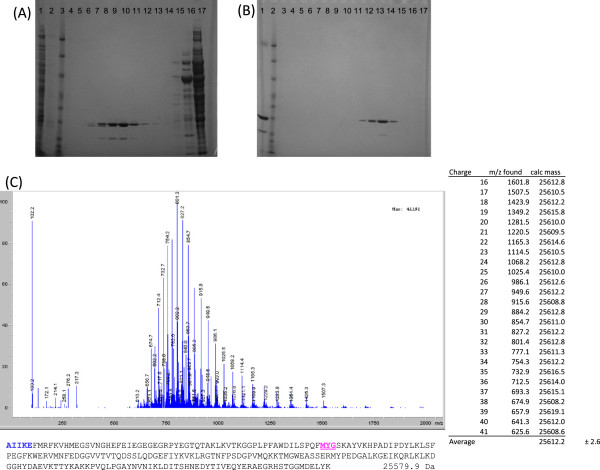
**Purification and mass determination of mCherry. (A)** 4-12% Bis-Tris SDS-PAGE gel of Q-sepharose purification. Lane 1 - cleared cell-free extract; Lane 2- wash; Lane 3 - Benchmark ladder; Lane 4 to17 - gradient elutions. **(B)** 4-12% Bis-Tris SDS-PAGE of size exclusion purification. Lane 1- loaded protein; Lane 2 - Benchmark ladder; Lanes 3 to 17 - elution fractions. **(C)** ESI-MS ion envelope of purified mCherry protein, deconvolution calculation, and primary sequence of mCherry protein based on observed mass and protein sequencing. Amino-acids detected from N-terminal protein sequencing in bold, chromophore forming residues underlined, with the expected mass of the mature protein listed after the primary sequence.

The mutagenesis study suggested that mCherry could be expressed as a functional 226 amino acid protein from pCherry29, but did not exclude the possibility that it was expressed as a longer protein form the pCherry10 plasmid. In order to address this, we purified mCherry from an *M. tuberculosis* transformant carrying pCherry10. Mass determination suggested that the protein had a size of 25612.2 Da, which closely approximated the predicted size for the 226 amino acid protein of 25579.9 Da (Figure 3). N-terminal sequencing confirmed that the purified protein commenced with the amino acids AAIKE, confirming that mCherry226 is the functional species of protein found in mycobacteria.

## Conclusions

We obtained high-level expression of mCherry from the G13 promoter in *M. tuberculosi*s. Analysis of the mature protein expressed in *M. tuberculosis* confirmed that the translational start site at +28 was utilized and the N-terminal sequence of the mature protein was AIIKE. The expression of truncated mCherry gave rise to highly fluorescent colonies, confirming that this truncated protein is functional. However, the absence of the GFP-derived peptide designed to allow efficient N-terminal fusions could have a negative impact on stability and function of fusion proteins.

## Methods

### Bacterial culture

*Escherichia coli* DH5α was cultured in LB medium or on LA agar. *M. tuberculosis* H37Rv was grown in Middlebrook 7H9 medium plus 10% v/v OADC (oleic acid, albumen, dextrose, catalase) supplement (Becton Dickinson) and 0.05% w/v Tween 80 or on Middlebrook 7H10 agar (Becton Dickinson) plus 10% v/v OADC. Hygromycin was used at 100 μg/ml where required.

### Construction of expression vectors

The mCherry expression vector pCherry10 was used as a template for mutagenesis [13]. Site directed mutagenesis was used to introduce a stop codon using primer pair CherrySTOPA1 5′- TCC ATG GTC TCG AAG TGA GAG GAG GAC AAC ATG-3′ and CherrySTOPA2 5′- CAT GTT GTC CTC CTC TCA CTT CGA GAC CAT GGA -3′ to generate pCherry29, or primer pair CherrySTOPB1 5′-GAC AAC ATG GCG ATC TGA AAG GAG TTC ATG CGC-3′ and CherrySTOPB2 5′- GCG CAT GAA CTC CTT TCA GAT CGC CAT GTT GTC-3′ to generate pCherry30. Stop codons are in bold.

### Quantitation of fluorescence in whole cells

*M. tuberculosis* was electroporated as described previously [25] and transformants selected with hygromycin. *E. coli* and *M. tuberculosis* were grown to stationary phase, harvested, washed twice in 10 mM Tris pH 8.0 and resuspended in 10 mM Tris pH 8.0 to an OD_580_ of 0.25, 0.10, 0.05 and 0.01 in 12 × 100 mm glass culture tubes. Fluorescence was measured on a Shimadzu RF-1501 spectrofluorimeter (Shimadzu) with a detection range of 0–1015 relative fluorescent units at the emission and excitation wavelengths of 587/610 nm.

### Western analysis of fluorescent proteins

Cell extracts were prepared from liquid cultures. Cells were harvested by centrifugation, washed twice in 10 mM Tris (pH 8.0), resuspended in 1 mL of 10 mM Tris (pH 8.0), and added to lysing matrix B tubes (QBiogene). Cells were disrupted using the Fastprep (QBiogene) set at speed 6.0 for 30 seconds. Samples were centrifuged for two min, and the supernatant was recovered and filter sterilized. Protein was quantified using a BCA kit (Pierce), and 10 μg of total protein was subjected to Western blot using rabbit anti-mCherry antibody (Clontech). The primary antibody was detected using horseradish peroxidase goat-anti-rabbit (Sigma), and activity was detected using an ECL kit (GE Healthcare).

### Mass determination and N-terminal sequencing of mCherry

Cell-free extracts were prepared from recombinant *M. tuberculosis* carrying plasmid pCherry10, diluted 5 fold into 20 mM Tris pH 8.0, 10 mM NaCl and loaded onto a buffer equilibrated Q-sepharose column (GE Healthcare). The column was washed with 10 column volumes of 20 mM Tris pH 8.0, 10 mM NaCl and mCherry protein was eluted using a stepwise increase in salt concentration up to 1 M NaCl. Eluted fractions were concentrated separately using Amicon Ultra Centrifugal Filters 10,000 MWCO (Millipore), coloured fractions pooled, and concentrated to 100 μL. Protein was further purified by applying pooled fractions to a Superdex 75 size-exclusion column (GE Healthcare) and eluting with 20 mM Tris pH 8.0, 10 mM NaCl buffer. Coloured fractions were pooled and concentrated to 25 μM (0.62 mg/mL). Protein was analyzed by LC-ESI-MS by diluting 15 μL of concentrate into 15 μL acetonitrile, 0.1% TFA, loading onto a Polaris 3 C8-A 150x4.6 mm column and eluting with an acetonitrile gradient over 30 min. Mass peaks from multiply charged species were deconvoluted to yield the mass of the mCherry protein. Edman degradation was carried out on 400 pmol of purified protein by Biosynthesis, Inc. on a Procise II Protein Sequencing System (Applied Biosystems).

## Competing interests

The authors declare that they have no competing interests.

## Authors’ contributions

PC, JM, EM and MF conducted the experimental work. PC, JM, EM and TP analyzed the data. TP, EM and PC wrote the paper. JM and MF provided input for the paper. All authors read and approved the final manuscript.
